# An Incarcerated Inguinal Hernia Initially Misdiagnosed as a Spermatic Cord Hydrocele: An Educational Case Report

**DOI:** 10.7759/cureus.92593

**Published:** 2025-09-17

**Authors:** Manami Oki, Ayami Sato, Mitsuki Hayashi, Ryo Ichibayashi

**Affiliations:** 1 Division of Emergency Medicine, Department of Internal Medicine, Toho University Sakura Medical Center, Chiba, JPN; 2 Department of Surgery, Toho University Sakura Medical Center, Chiba, JPN

**Keywords:** computed tomography, diagnostic imaging, inguinal hernia, misdiagnosis, spermatic cord hydrocele

## Abstract

Spermatic cord hydrocele and inguinal hernia are both common causes of groin swelling in adult males, but their coexistence is rare and may lead to diagnostic challenges.

A 59-year-old man previously diagnosed with right spermatic cord hydrocele on magnetic resonance imaging (MRI) presented with new-onset right groin pain and swelling. The initial evaluation suggested worsening of the spermatic cord hydrocele symptoms. However, contrast-enhanced computed tomography (CECT) revealed an incarcerated right inguinal hernia containing bowel and adipose tissue. Manual reduction was successful, and subsequent imaging confirmed resolution. MRI also showed a 10-mm mass consistent with a spermatic cord structure. Elective laparoscopic hernia repair (transabdominal preperitoneal approach (TAPP)) identified a right external inguinal hernia, and a multilocular hydrocele was also found adjacent to the hernia orifice, and the hydrocele was treated with suction and cauterization.

This case highlights the importance of reassessing initial diagnoses when new symptoms arise. A variety of imaging approaches are necessary to differentiate between overlapping groin lesions accurately. Clinicians should consider the possibility of a secondary pathology, such as a hernia, when managing a previously diagnosed spermatic cord hydrocele with symptom progression.

## Introduction

Spermatic cord hydrocele is a cystic lesion resulting from incomplete closure or obstruction of the processus vaginalis. It is anatomically classified into two types: the cystic type, which has no communication with the peritoneal cavity, and the funicular (chordal) type, which communicates with the peritoneal cavity [1,2]. In contrast, an inguinal hernia is defined as the protrusion of abdominal contents through the inguinal canal. Both conditions often present as groin masses, but inguinal hernias may enlarge with increased intra-abdominal pressure or postural changes and are often reducible, whereas spermatic cord hydroceles tend to be fluctuant, transilluminate on light examination, and are generally non-reducible. Imaging is often required for confirmation.

The relationship between spermatic cord hydrocele and inguinal hernia in adults remains unclear. In middle-aged and elderly men, there have been cases where it was difficult to differentiate between incarcerated inguinal hernia and spermatocele, and surgery revealed cystic spermatic cord hydrocele [3]. The incidence of men diagnosed with hydrocele or spermatocele is said to be approximately 100 per 100,000 men, but there are no reports showing the incidence of spermatic cord hydrocele itself, and it is considered rare [2,4].

Although anatomically and pathologically related, telling these conditions apart can be hard, especially when a new inguinal hernia appears after a previous diagnosis of an asymptomatic, non-communicating spermatic cord hydrocele. Not re-evaluating changing symptoms can lead to misdiagnosis or delayed treatment. So far, no cases have been reported where a patient first diagnosed with spermatic cord hydrocele later developed an incarcerated inguinal hernia.

This case highlights the diagnostic challenge in distinguishing between spermatic cord hydrocele and inguinal hernia, underscores the need for clinical re-evaluation when symptoms change, and emphasizes the essential role of definitive radiologic investigations, such as contrast-enhanced computed tomography (CECT) and MRI, particularly when clinical findings are equivocal.

## Case presentation

A 59-year-old man with no significant past medical or surgical history presented to the surgery department of another hospital five months earlier with right groin discomfort and swelling. The swelling was noted on the right side at the level of the pubic symphysis. As a result, an inguinal hernia was initially suspected, but due to difficulty with reduction, an MRI was performed. He was diagnosed with a right spermatic cord hydrocele. At the time, the patient reported only a small, painless groin swelling without tenderness or gastrointestinal symptoms. Therefore, conservative management without surgical intervention was selected.

On the day of his visit to our emergency department, the patient suddenly developed spontaneous pain in the right groin area. He also noticed that the previously palpable mass in the lower abdomen had increased in size. The groin pain was persistent and localized, without radiation. He reported no additional symptoms such as nausea, vomiting, fever, or changes in bowel habits. On arrival, he was alert and oriented, with stable vital signs: blood pressure 113/65 mmHg and pulse 75 beats/minute. He presented with a sudden onset of sharp, localized pain in the right groin that had persisted for several hours. The pain was non-radiating and constant in nature. On physical examination, a soft, oval-shaped mass approximately the size of a quail egg was palpable in the right inguinal region. The mass was slightly mobile and irreducible, with well-defined upper and lower margins. Its size remained unchanged with the patient in the supine position. The scrotum and testes were non-tender; however, groin pain was reproducibly elicited by scrotal traction. Abdominal examination revealed no abnormalities. Acetaminophen 1000 mg was administered, but provided only limited symptomatic relief.

**Figure 1:**
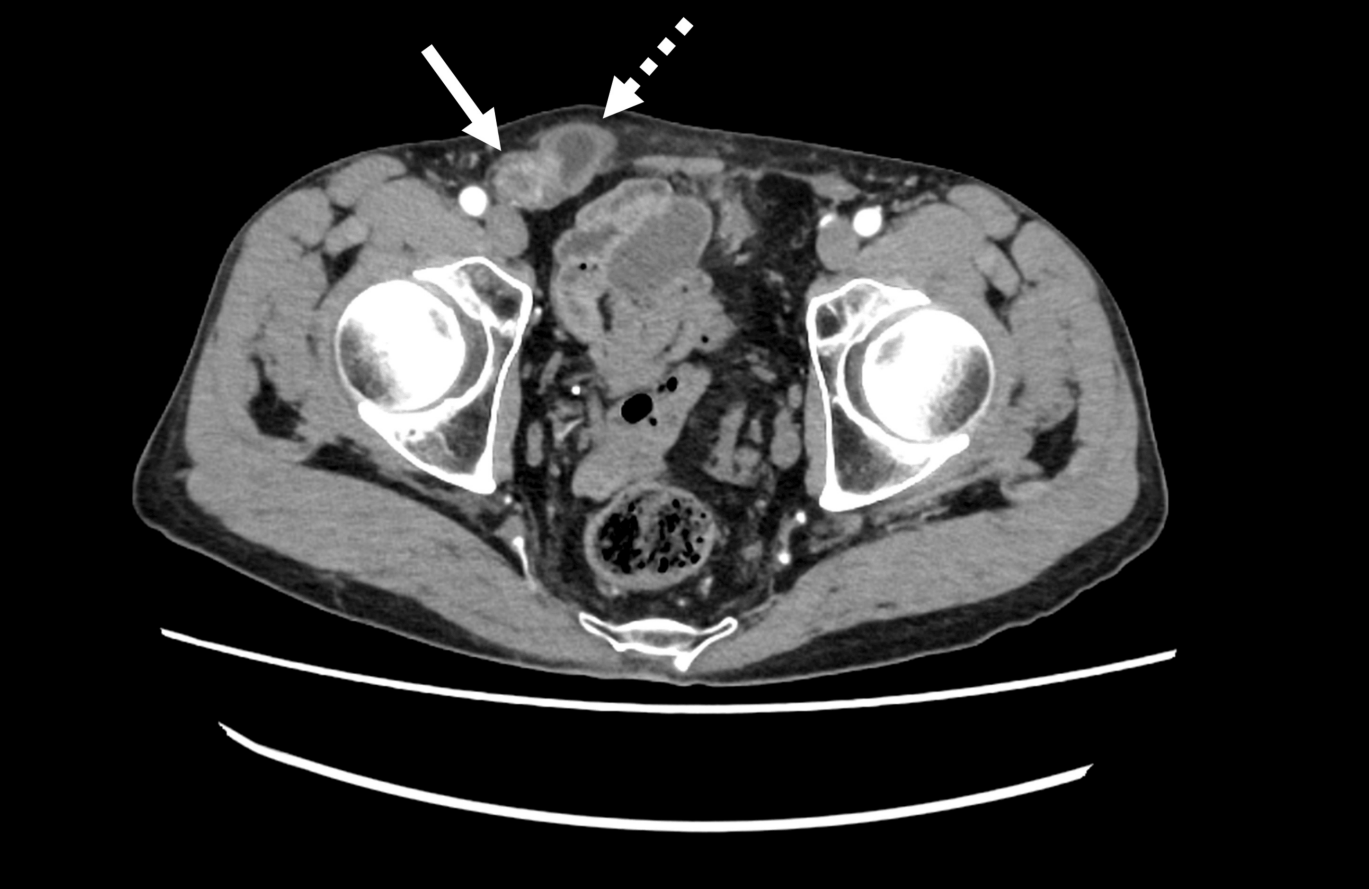
Abdominal contrast-enhanced computed tomography Axial contrast-enhanced computed tomography shows a spermatic cord mass (dotted white arrow) in the right inguinal region, along with herniated small bowel and fatty tissue (white arrow), indicating an incarcerated inguinal hernia.

While the previously diagnosed spermatic cord hydrocele was considered at first, the patient’s acute symptoms were inconsistent with the typical chronic progression of hydrocele and instead raised suspicion for a new diagnosis. To investigate further, an abdominal CECT was performed, revealing a spermatic cord hydrocele-like mass in the right groin, adjacent prolapsed bowel and adipose tissue, and dilated small intestine, findings consistent with an incarcerated right inguinal hernia causing intestinal obstruction (Figure 1).

**Figure 2:**
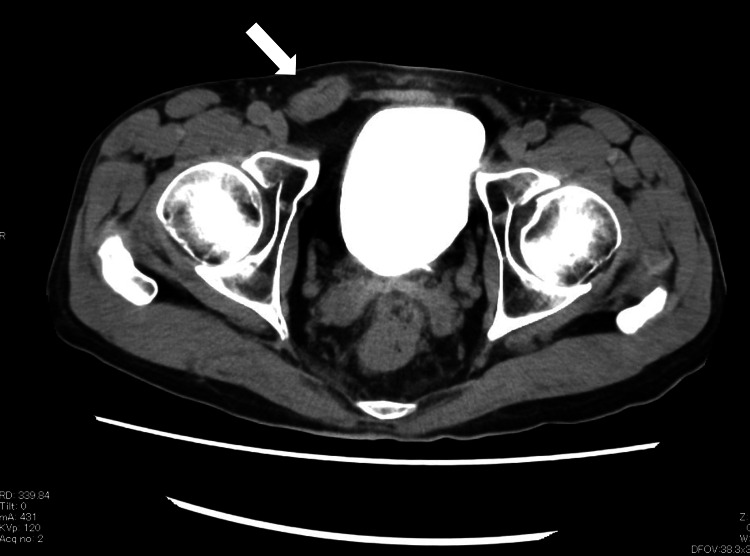
Post-reduction computed tomography An axial computed tomography image following manual reduction demonstrates localized edema along the spermatic cord in the right inguinal canal (white arrow), with no residual bowel incarceration.

The hernia defect measured approximately 1.5 cm. On presentation, the patient had persistent groin pain without abdominal distension, nausea, or vomiting. There were no signs suggestive of intestinal obstruction. As contrast enhancement of the bowel was preserved, manual reduction was attempted. Using ultrasound guidance, the intestine was identified within the hernial sac and successfully reduced. Follow-up CT confirmed resolution of the incarceration and persistence of a spermatic cord hydrocele (Figure 2).

The patient was hospitalized with bowel rest.

**Figure 3:**
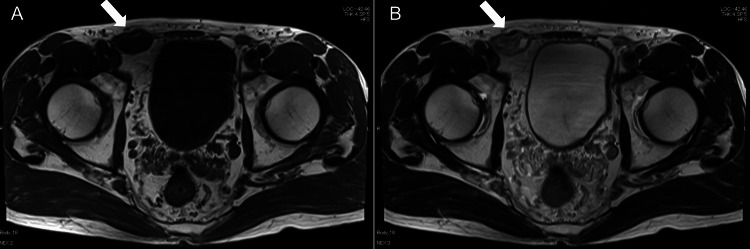
Magnetic resonance imaging of the groin The T1-weighted image (A) reveals a low-signal-intensity mass (white arrow) along the right spermatic cord, while the T2-weighted image (B) shows high signal intensity (white arrow). These signal characteristics suggest a cystic lesion.

On the following day, an MRI showed a 10 mm cystic lesion with fluid, in addition to the previously diagnosed spermatic cord hydrocele, suggesting a multilocular hydrocele. It showed that compared to the initial MRI, the spermatic cord hydrocele size remained almost unchanged (Figure 3).

By hospital day 2, bowel gas passage resumed, and an abdominal X-ray showed resolution of ileus; oral intake was restarted. The patient remained stable and was discharged on hospital day 3.

Elective laparoscopic hernia repair was subsequently performed using a transabdominal preperitoneal (TAPP) approach. After trocar insertion, the peritoneum was incised and the preperitoneal space was dissected. A 1.5 cm indirect inguinal hernia orifice was identified, along with a multilocular hydrocele on its ventral side. The hydrocele was aspirated and cauterized. A polypropylene mesh was placed to cover the defect, and the peritoneum was closed with continuous sutures. The postoperative course was uneventful, and the patient was discharged on postoperative day 3.

## Discussion

Spermatic cord hydrocele and inguinal hernia are both conditions that can present as groin masses. Because their clinical features often overlap, distinguishing between the two can be challenging, especially in obese adult patients. In particular, when an acquired inguinal hernia arises in a patient with a previously diagnosed non-communicating spermatic cord hydrocele, new symptoms such as swelling or pain may be misattributed to the progression of the hydrocele. This can result in delayed or missed diagnosis of the hernia, leading to potential complications such as bowel obstruction, strangulation, or necrosis. Although spermatic cord hydroceles are typically benign and asymptomatic, they can cause mass effect, infection, or diagnostic confusion when symptoms change. In our case, the assumption that the hydrocele was worsening delayed identification of the hernia. This highlights the importance of reassessing previous diagnoses in the face of new or atypical symptoms. To our knowledge, no previous reports have documented a patient initially diagnosed with a spermatic cord hydrocele who was later found to have an incarcerated inguinal hernia. This case illustrates a diagnostic pitfall and underscores the importance of clinical vigilance and the urgent need for timely re-evaluation.

In this case, the MRI initially diagnosed a spermatic cord hydrocele, and since the symptoms were mild, the patient was monitored. However, he later visited the emergency room due to new, spontaneous pain in the right groin and an enlarged mass. The differential diagnosis of an inguinal mass includes inguinal hernia, femoral hernia, varicocele, epididymitis, and testicular tumor [5]. The change in symptoms was initially thought to be a worsening of the spermatic cord hydrocele based on the existing diagnosis, but CECT revealed an incarcerated inguinal hernia with intestinal prolapse. The key point here is that the "appearance of new symptoms" was not just a progression of the existing condition but a sign of a new problem. Not sticking to the original diagnosis and actively reassessing when symptoms change can directly improve diagnostic accuracy and prevent serious complications.

Ultrasound examination is a non-invasive and straightforward method for the initial evaluation of spermatic cord hydrocele and inguinal hernia. However, when both conditions coexist, there are limitations to visualizing the disease [5,6]. One study evaluated the sensitivity and specificity of ultrasound, CT, and MRI in diagnosing hernias. Ultrasound was found to be the most accurate, but when investigating the sensitivity and specificity of diagnosing hernia types, it was shown that both CT and ultrasound may have high accuracy [6]. In this case, CECT confirmed the blood flow in the intestine, allowing safe manual reduction.

On the other hand, at present, there are no papers that specifically show the diagnostic rate of MRI for all inguinal masses (hernias, spermatic cord hydrocele, enlarged lymph nodes, etc.), but the diagnostic value of MRI in this case should not be underestimated. MRI is excellent at distinguishing between fat and water and shows high contrast in soft tissues, providing information that cannot be obtained with ultrasound or CT. Although most inguinal hernias can be diagnosed clinically and by ultrasound, MRI may play a complementary role in diagnosing cases that are clinically challenging or equivocal. This case reaffirms that multiple imaging modalities, including MRI when appropriately indicated, are beneficial for confirming complex inguinal lesions.

Later, laparoscopic inguinal hernia repair revealed a right indirect inguinal hernia and multilocular hydrocele ventral to the hernia orifice. Membrane rupture, aspiration, and coagulation procedures were performed. This suggests that some structures identified as spermatic cord hydrocele before surgery may be part of a more complex pathology near the hernia orifice, only fully revealed by intraoperative findings. Therefore, the importance of combining preoperative imaging with intraoperative findings should be emphasized.

The incidence of hydrocele or seminal vesicle hydrocele in men is estimated to be approximately 100 per 100,000, although there are no published reports documenting the incidence of spermatic cord hydrocele itself, and it is considered rare [2,4]. Treatment for spermatic cord hydrocele ranges from conservative to surgical [7]. Conservative management is appropriate for asymptomatic cases, while surgical intervention is recommended for persistent or increasing spermatic cord hydrocele [7]. Surgical options include open hydrocele resection or a laparoscopic approach, the latter of which is particularly useful in reducible cases [7]. In this case, laparoscopic inguinal hernia repair was performed, and intracystic aspiration was performed using the latter approach.

Treatment strategies vary for diseases that cause masses in the inguinal region, such as spermatic cord hydrocele, inguinal hernia, and hydrocele. Accurate diagnosis of inguinal diseases leads to appropriate treatment strategies, so when there is uncertainty about the diagnosis, a multifaceted imaging evaluation is necessary.

## Conclusions

This case highlights a diagnostic pitfall in adult groin masses. An acquired inguinal hernia can complicate a non-communicating spermatic cord hydrocele, and new pain or rapid enlargement may be misattributed to progression of the known lesion. In our patient, anchoring on the initial MRI diagnosis of spermatic cord hydrocele delayed reconsideration until CECT revealed an incarcerated inguinal hernia with intestinal prolapse. The key lesson is to prioritize flexible re-evaluation and repeat imaging when symptoms evolve, rather than relying on the initial label, to improve diagnostic accuracy, enable timely management, and prevent serious complications.
